# Second-Generation Pharmacological Chaperones: Beyond Inhibitors

**DOI:** 10.3390/molecules25143145

**Published:** 2020-07-09

**Authors:** My Lan Tran, Yves Génisson, Stéphanie Ballereau, Cécile Dehoux

**Affiliations:** SPCMIB, UMR5068 CNRS-Université Paul Sabatier-Toulouse III, 118 Route de Narbonne, F-31062 Toulouse, France; tran@chimie.ups-tlse.fr (M.L.T.); genisson@chimie.ups-tlse.fr (Y.G.)

**Keywords:** pharmacological chaperones, lysosomal storage disease, allosteric ligand, conformational disease, non-inhibitory chaperones

## Abstract

Protein misfolding induced by missense mutations is the source of hundreds of conformational diseases. The cell quality control may eliminate nascent misfolded proteins, such as enzymes, and a pathological loss-of-function may result from their early degradation. Since the proof of concept in the 2000s, the bioinspired pharmacological chaperone therapy became a relevant low-molecular-weight compound strategy against conformational diseases. The first-generation pharmacological chaperones were competitive inhibitors of mutant enzymes. Counterintuitively, in binding to the active site, these inhibitors stabilize the proper folding of the mutated protein and partially rescue its cellular function. The main limitation of the first-generation pharmacological chaperones lies in the balance between enzyme activity enhancement and inhibition. Recent research efforts were directed towards the development of promising second-generation pharmacological chaperones. These non-inhibitory ligands, targeting previously unknown binding pockets, limit the risk of adverse enzymatic inhibition. Their pharmacophore identification is however challenging and likely requires a massive screening-based approach. This review focuses on second-generation chaperones designed to restore the cellular activity of misfolded enzymes. It intends to highlight, for a selected set of rare inherited metabolic disorders, the strategies implemented to identify and develop these pharmacologically relevant small organic molecules as potential drug candidates.

## 1. Introduction

The tridimensional architecture of proteins, determined by their linear amino acid sequence, is essential to their native biological properties, trafficking and functions. Proper protein folding is thus a highly regulated process. For secretory or membrane-associated proteins, the folding process takes place in the endoplasmic reticulum (ER), while folding of cytosolic proteins takes place in cytosol. Yet, upon environmental and metabolic stress, or due to missense mutations, misfolded proteins may accumulate within cells leading to dysregulated cell phenotypes at the origin of hundreds of different protein conformational diseases [1].

Protein folding, along with protein synthesis, disaggregation, trafficking and degradation, is monitored by the proteostasis (protein homeostasis) network [2]. Proteostasis network is in charge of the integrity of the 10,000 to 20,000 distinct proteins forming the cellular proteome. This complex machinery acts as a quality control system monitoring nascent misfolded proteins. It operates not only at the ER, but also throughout different cell organelles as well as at the plasma membrane and in the extracellular fluid [3,4]. Molecular chaperone proteins are key players of proteostasis network that are up-regulated upon heat shock and unfolded protein responses. This large family of more than 300 highly conserved proteins is able to assist misfolded proteins refolding through unspecific cooperative macromolecular interactions. Other molecular chaperones can also prevent misfolded proteins aggregation and promote their degradation by the ubiquitin-proteasome system or autophagy [5]. The continuous degradation of a misfolded protein, such as a receptor, a transporter or an enzyme, may cause a loss-of-function disease. The large family of Lysosomal Storage Diseases (LSDs), caused by the burden in undegraded substrate of a deficient catabolic enzyme, is representative of this type of metabolic disorders. On the other hand, a decreased proteostasis network refolding capacity, favored by individuals aging, may lead to toxic misfolded protein aggregates and a subsequent gain-of-function molecular phenotype [6,7]. Neurodegenerative pathologies like Parkinson, Alzheimer and Huntington diseases fall into this category [8,9].

The biological concept of molecular chaperoning may be applied to help misfolded mutant proteins that retain some level of functionality escaping the cell quality control mechanism. Despite of its intrinsic pharmacological limitations, in particular due to its lack of protein specificity, this concept has inspired several chemical approaches for protein chaperoning. Besides the use of proteostasis regulators, they represent promising low-molecular-weight compound strategies to treat conformational diseases.

The first approach mainly deals with small organic osmolytes, referred to as chemical chaperones. These chemicals are trivial compounds, like glycerol, arginine, dimethyl sulfoxide, and trimethylamine *N*-oxide, used to modify the solvent conditions. Minimizing the protein-water interface, chemical chaperones frequently do not directly bind to the misfolded protein but rather promote their proper folding in destabilizing their unfolded form [10]. Sodium 4-phenylbutyrate (PBA), an otherwise approved drug for urea cycle disorders, proved to be a clinically relevant chemical chaperone restoring chloride transport of the mutated ΔF508 Cystic Fibrosis Transmembrane Conductance Regulator (CFTR) [11]. PBA would however both act as a hydrophobic chemical chaperone [10] and a heat shock protein inducer. Overall, the widespread therapeutic application of chemical chaperones appears to be hampered by the high cell concentrations needed, their lack of specificity and of controlled systemic bioavailability, potentially giving undesired effects.

Pharmacological chaperones (PCs), also called pharmacoperones or pharmacochaperones, are the basis of the second main chemical approach, introduced in the early 2000′s [12,13]. Unlike chemical chaperones, PCs are specific ligands that stabilize the correct conformation of misfolded proteins by selectively binding to them. In doing so, they escort the mutant protein to its normal cell location where it may express its residual level of function. Quite naturally, this strategy historically took advantage of the available portfolio of high affinity competitive enzyme inhibitors. These active-site specific chaperones, paradoxically used to restore the function of mutant proteins whereas they were designed to inhibit their wild-type counterpart, constitute the “first-generation pharmacoperones”. Moreover, these first-generation pharmacoperones are not limited to the pair “enzyme/competitive inhibitor”. This concept has also been extended to orthosteric ligands for receptors [14] as well as correctors for transporters, such as CFTR [15]. Many studies demonstrated the potential of these first-generation PCs to thermodynamically stabilize proper protein folding at rather low concentration. In order to restore protein activity, the PC has however to be ultimately displaced by the endogenous protein ligand. If most of the reversible inhibitors showed significant *in vitro* enhancement in protein activity, it was not necessarily the case *in vivo*. Isofagomine (Plicera^TM^), a glucocerebrosidase competitive inhibitor developed as a PC against the LSD Gaucher disease, failed in phase II clinical trial for lack of efficacy (see Section 2.1). An important credit was given to the use of first-generation PC with the approval in 2018 of the seminal α-galactosidase A competitive inhibitor 1-deoxygalactonojirimycin (Galafold^TM^, migalastat) against the other LSD Fabry disease (see Section 2.2) [16]. Yet, since the protein inhibition by first-generation PCs is not directly correlated to their chaperoning activity, it remains clinically difficult to adjust their dosage regimen so as to control the balance between those two contradictory effects.

Non-inhibitory PCs, considered as the “second-generation pharmacoperones”, have emerged in order to circumvent the issues associated to the protein chaperoning with competitive inhibitors. In selectively binding to another pocket than the active site, these promising PCs present the advantage of potentially activating the misfolded protein without interfering with its active site, thus minimizing the risk of adverse competition with the endogenous substrate [6,17,18]. In addition, since most of the targeted enzymes are glycoside hydrolases, especially for LSDs, moving away from glycomimetic inhibitors offers opportunities to develop more specific ligands. However, unlike inhibitors, that are rather straightforward to develop with standard enzymatic assays using endogenous substrate-derived probes, the search for such ligand is by far more demanding. Indeed, by definition, neither the druggable pocket nor a reference binder is *a priori* known and pharmacophore identification likely requires a massive, *in silico* and/or *in vitro*, screening-based approach. This issue may explain why, so far, only a few second-generation PCs have been developed. Beyond sapropterin, a phenylalanine hydroxylase endogenous ligand used since 2007 in the treatment of phenylketonuria (see Section 3.1), the anti-aggregative protein binder tafamadis was approved in 2012 against the neurodegenerative disease transthyretin-related hereditary amyloidosis [19].

Since the appearance of the concept of PC [12,13], among hundreds of research articles referencing this therapeutic approach, more than sixty percent deal with enzymopathies [6]. Many review articles were also published, either focusing on a specific pathology [17,18,20], a specific target [14,21] or stressing on a specific point of view [22,23]. We dedicated the present literature survey to non-inhibitory PCs, regarded as the “second-generation pharmacoperones”, and we concentrated our attention on enzymes since they represent the large majority of the PC targets. This review article, focusing on the most studied enzymatic targets at the origin of inborn errors of metabolism, is thus organized according to the related conformational diseases. In light of the strong potential of second-generation PCs, the discovery, evaluation and potential applications of non-inhibitory ligands as conformational effectors for the functional rescue of pathological misfolded proteins is thereafter detailed.

We implemented the following bibliographic methodology to survey the literature. Three bibliographic databases were used: Scifinder (https://scifinder.cas.org), Web of Science (https://apps.webofknowledge.com) and Pubmed (https://www.ncbi.nlm.nih.gov/pubmed/). The chosen keywords were: “pharmacological chaperones” and “enzyme”. From the obtained references, we included those mentioning non-inhibitory or weak inhibitory pharmacological chaperones and we excluded those describing inhibitory pharmacological chaperones or pharmacological chaperones without explicit inhibition data.

## 2. Second-Generation Pharmacological Chaperones against LSDs

Complex metabolites are degraded in the lysosome, an acidic cell compartment dedicated to catabolism. Within this organelle, soluble hydrolases chop macromolecules off into monomeric components, allowing expulsion of toxic products or metabolic recycling of reusable substrates [24]. The progressive built up in substrate burden resulting from a specific catabolic enzyme deficiency is at the origin of a LSD. Although this heterogeneous group of more than 50 rare genetic diseases can have various origins, the misfolding of a nascent enzyme in the ER is a key determining factor of LSDs. Impairment of protein trafficking to lysosome following its interception by quality control system is responsible for the pathological phenotype [25]. LSDs were part of the first genetic diseases for which biochemical bases were elucidated, even though their pathogenic mechanisms remain yet to be fully clarified [26].

A great part of the LSD pathological symptoms is currently cured by enzyme replacement therapy (ERT) consisting in the periodic infusion of recombinant enzymes. This approach is however notably expensive and inefficient for neurological symptoms. ERT is in addition susceptible to trigger an immune response against the administrated enzyme. Substrate reduction therapy (SRT) is also a validated strategy based on the inhibition of the enzyme catalyzing the biosynthesis of the accumulated metabolite. However, this approach, requiring the selective targeting of a specific transferase, has so far only shown clinical results for Gaucher and Niemann-Pick type C diseases [27]. Finally, PCs were introduced as potential drugs based on the notion that a restoration of only 10 to 30% of normal catalytic activity was sufficient to prevent pathological manifestations [28,29,30,31,32]. To date, only Fabry disease has clinically benefit from this strategy with the use of a first-generation PC (see Section 2.2). In the case of LSDs, PC may also be used to overcome the limitations of the ERT. Thus, Amicus Therapeutics is currently performing a clinical trial with a combined PC/ERT therapy against Pompe disease (Section 2.3). Two types of LSDs are reviewed thereafter, sphingolipidosis such as Gaucher, Fabry and Krabbe diseases, which is one of the major group of LSDs resulting from the burden of sphingolipids in lysosomes and glycogenosis, such as Pompe disease, a minor group ensuing a glycogen accumulation.

### 2.1. Gaucher Disease

Gaucher disease (GD, OMIM #231000) is one of the most common sphingolipidosis, with a variable incidence depending on the population studied. Indeed, the average prevalence is 1:60,000 births but it can reach 1:800 in the Ashkenazi Jewish population [33]. This inherited disorder is caused by mutations in the *GBA1* gene, leading to the deficiency in the enzyme β-glucocerebrosidase (Gcase, EC 3.2.1.45), also called glucosylceramidase or acid β-glucosidase. The misfolded Gcase, unable to properly hydrolyze glucosylceramide (GlcCer) in macrophages of spleen, liver and bone marrow, leads to 3 phenotypes depending on GlcCer accumulation rate and age of patients. Type 1 GD (GD1), the most common (90–95%) and also the least severe manifestation, is characterized by variable physical symptoms such as splenomegaly, hepatomegaly and bone pain but also hematologic symptoms. Type 2 GD (GD2), the most severe form, is distinguished by acute neurological involvements at an early age leading to death. Type 3 GD (GD3), is a heterogeneous phenotype characterized by more or less severe neurological impairments, developed at a later age than type 2 and can be associated with some of type 1 symptoms. To date, only GD1 can be cured by ERT (imiglucerase) as a first-line treatment or by SRT with an oral treatment alternative (miglustat or eliglustat) [34]. Among the 350 mutations reported for GD (from the Human Gene Mutation Database (HGMD): http://www.hgmd.cf.ac.uk/ac/index.php), the most common are p.N370S (53%), associated with GD1 symptoms and p.L444P (18%) often involved with neurological signs [35]. Many reversible competitive inhibitors used as PC have been studied but most of them were only targeting p.N370S [10,36]. The iminosugar isofagomine (Plicera^TM^) is a first-generation PC abandoned for inefficiency after phase II clinical trials [37]. The expectorant ambroxol, identified as a mixed-type Gcase inhibitor among a collection of FDA-approved drugs, proved to be a promising PC for GD [38]. Since it is also effective in Fabry and Pompe diseases when used in synergy with the corresponding galactose or glucose mimetics 1-deoxygalactonojirimycin (Galafold^TM^, migalastat) (see Section 2.2) and 1-deoxynojirimycin (duvoglustat, AT2220) (see Section 2.3), ambroxol is however believed to act through a more complex mechanism [39].

It is worth noting that a close link between Gaucher and Parkinson diseases has been established*: GBA1* gene mutations are the highest risk factor for Parkinson disease [40,41]. Although several explanations of the connection between *GBA1* gene mutations and Parkinson disease onset were stated [42], the mechanism remains unclear. Yet, observation that Gcase activation leads to α-synuclein decrease makes PC of Gcase promising drugs also for Parkinson disease patients. 

In 2007, a quantitative high throughput screening (qHTS) campaign was initiated by the NIH. Starting from the observation that iminosugar derivatives are non-specific and have relatively short half-lives in cells [43], Sidransky, Austin and coll. developed an HTS to uncover new non-sugar small-molecule Gcase PCs both as research tools and as starting points for the development of new GD therapies [44,45]. With this wild type-Gcase targeting HTS, several novel inhibitory compounds were found as potential PCs [44,46,47] but no promising enzyme activators were identified. Another drawback of this HTS was the fact that these novel inhibitors showed reduced potency in confirmatory cell-based assays. In 2012, to circumvent this drawback, the same group implemented a new qHTS on mutant enzyme extracted from the spleen of a GD patient with genotype p.N370S/p.N370S [48]. Indeed, Gcase extracted from tissues, being present with the native activator saposin C and other potential cofactors, is found in a more native physiological environment. From the screening of a library of 250,000 compounds, thirty new lead activators were identified. Among these compounds 2-(2-((4-bromophenyl)amino)-2-oxoethoxy)-*N*-(2-(methyl(phenyl)amino)-2-oxoethyl)benzamide (ML266 (or NCGC00182186), Table 1), a salicylic acid derivative, and *N*-(4-ethynylphenyl)-5,7-dimethylpyrazolo[1,5-*a*]pyrimidine-3-carboxamide (ML198 (or NCGC00188758), Table 1), a pyrazolopyrimidine carboxamide derivative, were selected as representative of each series [49]. These PCs, while showing no Gcase inhibition, were capable of increasing enzyme activity in p.N370S and p.L444P cell-based assays using patient-derived fibroblasts. They also proved selective toward Gcase compared to α-glucosidase and α-galactosidase.

In 2016, Aflaki et al., studied 2-[2-[(4-iodophenyl)amino]-2-oxoethoxy]-*N*-[2- (methyl-phenylamino)-2-oxoethyl]-benzamide (NCGC607, Table 1), a non-inhibitory PC, salicylic acid derivative analog of ML266 [50]. Since it is difficult to evaluate drugs activity on neuron, the use of induced human pluripotent stem cells (iPSCs) interestingly allows validating a larger number of small molecule drugs. iPSCs were generated from fibroblast lines of GD1, GD2 and GD1-Parkinson disease patients and differentiated into macrophages (iMacs) and dopaminergic neurons (iDA). Immunofluorescence and Western Blot assays showed that NCGC607 is able to increase Gcase activity, improve Gcase translocation, decrease GlcCer accumulation and enhance pathological α-synuclein clearance for GD1, GD2 and GD-Parkinson disease in both iMacs and iDA. Interestingly, the most significant increase in Gcase activity and α-synuclein clearance was observed for GD1-Parkinson disease and GD2 which are promising outcomes for Parkinson but also neuropathic form of GD [51].

For pyrazolopyrimidine derivatives, structure-activity relationship and biological data confirmed ML198 as the most potent non-inhibitory PC with a chaperoning activity. *In vitro* and *in vivo* ADME (Absorption, Distribution, Metabolism, Excretion) studies were performed and showed good stability, oral absorption but also capacity to cross the blood-brain barrier [52]. Cell assays were also carried out in iMacs generated from GD1 and GD2 patient’s fibroblast [53] and from patients with different Parkinson disease-linked mutations [54]. ML198 was shown to enhance Gcase activity in lysosomes and decrease α-synuclein in several synucleinopathy models.

Another pyrazolopyrimidine, LTI-291 (5,7-dimethyl-*N*-((1*r*,4*r*)-4-(pentyloxy)cyclohexyl)-pyrazolo[1,5-*a*]pyrimidine-3-carboxamide, Table 1), is the first and only small-molecule allosteric activator of Gcase which is currently tested in phase 2a clinical trials for GD-Parkinson patients held by Lysosomal Therapeutics Inc. [55,56,57]. To date, phase 1 clinical trials proved the safety of LTI-291 and its capacity to accumulate up to 1% in healthy patient brain corresponding to 1 µM of the drug which is sufficient to double Gcase activity [58].

These pyrazolopyrimidine as well as pyrrolopyrimidine derivatives were largely explored by Krainc and coll. leading to recent patent filings [59,60].

Quinazoline derivatives were identified as non-sugar inhibitory PCs of Gcase from several HTS [46,61,62]. The recent co-crystallization of the Gcase with a quinazoline derivative not only helped to understand the mechanism of non-iminosugar inhibitory PC but also allowed the discovery of an allosteric binding site which could be responsive to activators (PDB code 5LVX) [63]. Thanks to a broad structure-activity relationship study in this compound series [62,64], Silvermann et al., recently found that *N*-methylation of some of the quinazoline inhibitors derivatives turned them into activators. Compound S-181 ((*S*)-*N*-((2,3-dihydrobenzo[b][1,4]dioxin-2-yl)methyl)-*N*-methyl-2-(pyridin-3-yl)quinazolin-4-amine, Table 1) was highlighted as the most potent non-inhibitory Gcase PC with specificity towards the Gcase and not α-glucosidases or galactosidases [64]. Treatment with S-181 (15 µM) increased Gcase activity in p.N370S and p.L444P fibroblasts and also in iPSC-derived dopaminergic neurons from Parkinson patients [65]. Moreover, intraperitoneal single administration of S-181 (50 mg/kg) to wild-type mice and a mouse model of GD-related synucleinopathy activated the Gcase activity, reduced GlcCer accumulation and reduced the amount of insoluble α-synuclein in brain tissue, proving the CNS penetrance. In light of these preliminary results, S-181 reveals an interesting candidate for treating neuropathic GD and Parkinson disease.

Several promising Gcase non-inhibitory PCs have been uncovered and investigated for GD1, GD2 but also Parkinson disease and further pharmaco-kinetic and -dynamic studies are needed to develop the most effective analogs as well as mechanistic studies in order to identify the allosteric binding sites involved. 

### 2.2. Fabry Disease

Fabry disease (FD, OMIM #301500) is due to mutations in the X-linked gene *GLA* encoding for the lysosomal α-galactosidase A (α-Gal A, EC 3.2.1.22). More than 800 different *GLA* mutations are recorded, including almost 590 missense/nonsense mutations (from HGMD). The glycosphingolipids burden caused by the enzyme malfunction induces several cell components dysfunctions, ultimately resulting in oxidative stress, inflammation and apoptosis [66]. Male hemizygous patients display the most common symptoms, but female heterozygous can also suffer from severe forms. This systemic disease is associated to progressive renal failure, cardiac and cerebrovascular disorders, peripheral neuropathy and skin lesions, among other abnormalities. In some cases, late-onset milder phenotypes can affect only a small set of organs, like the heart or kidneys. Fabry patients survive into adulthood but exhibit lifespan shortened by 10 years, for females, to 20 years for males [67]. Recent data report a highly variable disease prevalence ranging from 1:8454 [68] up to 1:1250 [69], depending on the region of the globe. Yet, FD has been estimated to account for 8.8% of the patients presenting inherited errors of metabolism, as compared to 4.2% for GD, and is regarded as the most common LSD [70].

α-Gal A is responsible for the lysosomal hydrolysis of terminal α-1,2- and α-1,3-linked galactosyl residues from various glycoconjugates. Its reduced catalytic activity causes mainly accumulation of globotriaosylceramide (Gb3) and as well as related deacylated lyso-analogues that may serve as biomarkers for therapeutic efficacy assessment. The dimeric protein is composed of two monomers embedding an active site located in the *N*-terminal domain and containing two catalytic aspartate residues [71]. Each monomer also bears several *N-*linked glycosylation sites allowing presentation of mannose 6-phosphate residues essential to protein cellular addressing.

Two therapeutic options are currently available against FD. The ERT is based on biweekly intravenous infusions of the recombinant enzymes agalsidase alfa (Replagal^TM^) or agalsidase beta (Fabrazyme^TM^). As an alternative to this time-consuming and expensive approach, other less constraining therapeutic options were sought. Providing that patients display amenable mutations with residual catalytic activity, enzyme folding stabilization by a PC proved to be a relevant strategy. The seminal iminosugar-based galactose mimetic l-deoxygalactonojirimycin (DGJ, Galafold^TM^, migalastat) commercialized for oral therapy is however also a competitive enzymatic inhibitor requiring an intermittent administration to be effective. Limitations of PCs presenting an adverse inhibitory effect prompted several alternative strategies, such as the use of DGJ in synergy with the approved drug ambroxol [39], or the search for α-Gal A non-inhibitory allosteric effectors.

Pérez-Sánchez et al., reported in 2016 the virtual screening-based identification of an α-Gal A allosteric hotspot and protein ligands thereof [72]. *In silico* calculations were run with a random subset of 9 million compounds from the ZINC database. Among them, 10,000 purchasable molecules were selected for docking on the basis of their similarity with α-d-galactose. The crystal structure bound to α-d-galactose in the active site and β-d-galactose in a secondary α-Gal A site was used (PDB code 3S5Z). Docking simulations allowed identification of a druggable pocket in proximity to the α-Gal A site and with no overlap with the active site. In the lack of any approved drug, 2,6-dithiopurine (DTP) (Table 1) was selected as a biologically safe and site-selective ligand of this new allosteric site.

The absence of enzyme inhibition was verified with the recombinant wild-type protein (Fabrazyme^TM^) at the unusually high concentration of 6 mM. The ability of DTP to bind the wild-type α-Gal A was demonstrated in thermal shift assay at the same concentration. The ligand proved able to reversibly and selectively stabilize the enzyme towards thermal denaturation, either alone or in synergy with 40 µM of DGJ. Urea-induced protein unfolding experiments were also used to assess α-Gal A stabilization by DTP. The enzyme unfolds at 3 M in urea in the presence of the ligand vs. 2.7 M in its absence.

Regarding cell-based assays, the mutant p.A230T was first selected to be expressed in COS-7 cells since it is non-responsive to DGJ but retains a degree of functionality that renders it eligible for PC therapy. DTP was shown to rescue the deficient protein at concentrations down to 2 mM as evaluated by western blotting and enzymatic assay. This effect is significantly potentiated by the use of a one order of magnitude lower dosage in DGJ or galactose, but not ambroxol, whereas these established PCs alone do not promote p.A230T mutant maturation in COS-7 cells at standard concentrations. Cellular protein processing was also promoted by DTP for p.C56Y, p.C63Y and p.E341D mutants, yet without enzymatic activity recovery. Mutations of several protein residues in the allosteric site (p.A37T, p.P40S, p.M42T, p.M42V) proved to suppress the response to DTP in the same cellular model, in accordance with the binding of the ligand to the protein pocket identified by *in silico* docking. This work opens perspectives for lead optimization towards more potent and specific α-Gal A PC candidates potentially useful against FD.

### 2.3. Pompe Disease

Pompe disease (PD, OMIM #232300), also known as glycogen storage disease type 2 or acid maltase deficiencies, is caused by mutations of the gene coding for the lysosomal α-glucosidase (GAA, E.C.3.2.1.20). This enzyme catalyzes the hydrolysis the α-1,4 and 1,6-glycosidic bonds of glycogen to produce glucose in lysosomes. The incidence of PD is wide ranging from 1:14,000 (in African populations and African-Americans) to 1:238,000 in Europe [73]. There are more than 400 different GAA mutations producing PD symptoms (from HGMD). Most of these mutated proteins keep their enzymatic activity but suffer from inoperative trafficking to the lysosome. As GAA is the sole protein responsible for glycogen degradation in lysosomes, its functional deficiency causes accumulation of the glycogen in all tissues, especially in muscles and heart, giving rise to reduced motor functions and respiratory deficits. The resulting spectrum of pathological manifestations ranges from slow progressive phenotype with late onset to devastating childhood-onset [73,74].

From a therapeutic point of view, the ERT, often completed with artificial ventilation and physical therapy, remains the only treatment for PD. The recombinant *h*GAA produced in Chinese hamster ovary cells line was approved in 2006 (Myozyme™) and allowed the increase of patient’s survival as well as a slowdown in the disease progression. Despite these obvious benefits, the patient’s response to ERT is highly variable and the efficiency in treating the muscular involvement remains insufficient [73]. PC therapy was also explored against PD, either alone or in combination with ERT. The promising PC 1-deoxynojirimycin (duvoglustat, AT2220) failed in a phase II clinical trial because of serious adverse events (https://clinicaltrials.gov/ct2/show/NCT00688597). *N*-butyl-d-deoxynojirimycin (miglustat) also improved the residual activity and lysosomal trafficking of mutated GAA [75]. When co-administrated with recombinant GAA, *N*-butyl-d-deoxynojirimycin was found to increase enzyme physical stability and therapeutic efficacy [76,77]. Based on these results, Amicus Therapeutics is currently performing a clinical trial against PD with a therapeutic approach combining PC therapy and ERT.

In 2009, the NIH implemented an HTS screening [78] in order to identify novel non-iminosugar activators of GAA according to a strategy similar to the one developed against GD (see Section 2.1). Thanks to this HTS, Marugan et al., uncovered ML247 (or NCGC00183885, Table 1) as the first non-inhibitory PC of GAA [79,80].

In 2012, Parenti and coll. identified *N*-acetylcysteine (Table 1) as another PC with no GAA inhibitory properties [81] and showed that the latter enhanced the recombinant *h*GAA stability as a function of pH and temperature. In addition, an increase in residual activity of mutated GAA was observed in PD fibroblast and COS7 cells in presence of *N*-acetylcysteine. Relying on computational studies, authors anticipated the allosteric behavior of this PC. Recently high-resolution crystal structures of recombinant *h*GAA was reported [82]. Co-crystallization with chaperone *N*-acetylcysteine (PDB code 5NN4) revealed two allosteric binding sites and thus confirmed that *N*-acetylcysteine acts as an allosteric PC.

While being a PC of mutant and wild-type GAA, *N*-butyl-d-deoxynojirimycin also acts as an inhibitor of recombinant *h*GAA and various glycosidases [83]. In the course of their research on the impact of the stereochemistry on biomolecular recognition, D’Alonzo and coll. demonstrated that the enantiomer *N*-butyl-l-deoxynojirimycin (Table 1) enhanced lysosomal GAA levels in PD patients fibroblasts carrying the p.L552P/p.L552P mutation [83]. Interestingly no inhibition at 1 mM was found with *N-*butyl-l-deoxynojirimycin against 14 representative examples of various glycoside hydrolase families including recombinant *h*GAA. Thus, *N*-butyl-l-deoxynojirimycin is a non-inhibitory enhancer of GAA activity. Moreover, synergetic effects have been highlighted when it was co-incubated with recombinant *h*GAA. However, additional studies including for instance crystallographic data would be necessary to demonstrate the allosteric binding of *N*-butyl-l-deoxynojirimycin.

### 2.4. Krabbe Disease

Krabbe disease (KD, OMIM #245200), also known as globoid cell leukodystrophy, is an autosomal recessive LSD. KD is a sphingolipidosis due to mutations in the *GALC* gene encoding the lysosomal enzyme β-galactocerebrosidase (GALC, EC 3.2.1.46) which catabolizes the hydrolysis of galactose from galactocerebroside and galactosylsphingosine (psychosine). The accumulation of cytotoxic psychosine leads to apoptosis of oligodendrocytes and to demyelination of the central and peripheral nervous systems. KD includes early childhood-onset forms (85–90%) with a severe, rapidly progressive and fatal neurodegenerative disease evolution and late-onset (late infant / juvenile) and adult forms (10–15%) clinically more heterogeneous and less severe [84,85]. The median prevalence of KD is estimated to be 1:100,000 births with wide variations between countries [86,87].

For a child younger than age six months with early childhood-onset KD, treatment is supportive and focused on increasing the quality of life and avoiding complications [88]. For patients with a pre-symptomatic infant form and a mild late form, treatment is limited to the transplantation of hematopoietic stem cells, which slows the progression of the disease [89]. Other therapeutic options (PC therapy, ERT, gene therapy) are currently being studied in animal models [84]. Like for other LSDs, several iminosugar or aminocyclitol derivatives were identified as PC with inhibitory activity in the case of KD [22,90].

Among the 200 mutations reported to cause KD (from HGMD), the most common is a 30-kb deletion that encodes nonfunctional proteins responsible for the infantile phenotype. However, the majority of the other mutations associated with KD are missense mutations and most of these are predicted to disrupt the fold of enzyme and are as such candidate for chaperone therapy [20].

In 2010, Lee et al. [91] reported α-lobeline (Table 1) as the first PC for KD. Even if α-lobeline was identified through an *in vitro* enzymatic activity screening as a “relatively” weak inhibitor of GALC, it was shown to increase the GALC activity of the KD-associated hyperglycosylation mutation p.D528N in neuronal cells. Nevertheless, the high dose required to observe this chaperoning effect (240 µM) and the selectivity for a single mutation raise questions about the therapeutic potential of this compound.

A few years later, Berardi et al. [92] also implemented an enzymatic activity assay to screen several small molecules as potential PCs. At the tested concentration (400 µM), none of the molecules showed significant inhibition and two of them, α-lobeline and 3′,4′,7-trihydroxyisoflavone (Table 1) were selected for further investigations. The effect of these compounds was studied on several patient-derived fibroblast cell lines carrying missenses mutations. α-Lobeline and 3′,4′,7-trihydroxyisoflavone both increased the GALC activity in the cell lines homozygous for the p.G553R mutation and for the p.G57S mutation and in the cell line expressing both p.E130K and p.N295T mutations. When tested on mutations not predicted to affect the fold of GALC, no effect on the enzyme specific activity was observed for any of the two tested compounds. This specificity for mutations affecting the protein folding supports the fact that α-lobeline and 3′,4′,7-trihydroxyisoflavone act as PC on GALC. However, the *in silico* molecular docking analysis identified nine different binding sites on GALC for α-lobeline and 3′,4′,7-trihydroxyisoflavone which is consistent with a non-specific chemical chaperone behavior, which add to the fact that the 3′,4′,7-trihydroxyisoflavone is known to inhibit other cellular enzymes [93].

## 3. Non-Inhibitory Pharmacological Chaperones for Miscellaneous Conformational Diseases

### 3.1. Phenylketonuria

Phenylketonuria (PKU; OMIM #261600) is the most common amino acid metabolism disease and this inherited disorder concerns on average 1:10,000 new-borns [94]. PKU is due to mutations of the *PAH* gene encoding the hepatic enzyme phenyl hydroxylase (PAH, phenylalanine 4-monooxygenase; EC 1.14.16.1) which catalyses the para-hydroxylation of l-Phe to l-Tyr. To ensure its function, PAH needs a non-heme iron, (6*R*)-l-*erythro*-5,6,7,8-tetrahydrobiopterin (BH4) (Table 1) as a co-factor and dioxygen as a co-substrate. At present, more than 780 PKU-associated mutations have been reported for the *PAH* gene (from HGMD). In most cases, these mutations produce misfolding of the enzyme, its loss of function, and premature ubiquitin-dependent degradation. The subsequent decrease in PAH activity gives rise to the neurotoxic l-Phe accumulation in the blood and in the tissues. Untreated PKU may lead to profound and irreversible neurologic damages. To limit the PKU evolution, a restrictive protein-controlled diet is recommended all along patients’ life. For a subset of patients with specific *PAH* mutations, administration of BH4, formulated as sapropterin dihydrochloride (Kuvan™) can alleviate this diet [95].

The PAH activity is balanced by three main mechanisms, i.e., activation by l-Phe, activation by phosphorylation and BH4 negative regulation [96]. Activation of PAH by l-Phe occurs at high concentrations of l-Phe in blood while negative regulation by BH4 takes place at a low level. Co-crystallizations of PAH with BH4 or l-Phe allowed the fine understanding of these regulation mechanisms [97,98,99,100]. PAH protein exists as a mixture of native structures in “resting” or in “activated” states depending on the l-Phe concentration levels [97]. In the resting-state, l-Phe cannot easily fit into the active site while in the “activated” state, the active site is fully accessible. Conformational change between both states is induced by the binding of l-Phe to an allosteric site located in the *N*-terminal regulatory domain [101,102,103].

Several studies have attempted to explain the mechanism of action of BH4 which causes a decrease in blood phenylalanine concentrations [104,105]. It was reported that the co-factor BH4 have multifactorial effects on the wild-type and the mutated PAH, including their stabilization and the protection from inactivation and proteolytic degradation [106]. The PC behaviour of BH4 was highlighted by Erlandsen et al., on fifteen BH4-responsive mutants [107]. The recent co-crystallization of the full-length *h*PAH with BH4 was reported, helping to understand the stabilization by the cofactor (PDB code 6HYC) [98]. Authors showed that the stabilization of the protein by BH4 is initiated at its binding-site in the catalytic domain but also affects the whole structure of the protein. While the co-factor downregulates the protein at low l-Phe concentration, BH4 is not strictly speaking a PAH inhibitor and therefore can be classified as a non-inhibitory PC.

As the BH4 treatment cannot be extended to all mutations, there is a need to develop new PCs with wider applications. A screening using DSF (Differential Scanning Fluorimetry) allowed the identification of 5,6-dimethyl-3-(4-methyl-2-pyridinyl)-2-thioxo-2,3-dihydrothieno[2,3-*d*]pyrimidin-4(1*H*)-one as being able to increase the activity and stability of protein steady-state by promoting the tetrameric form [108]. Additional studies showed that this compound acts as a competitive inhibitor with respect to BH4. Few years later, Santos-Sierra et al. [109], performed a shape-focused virtual screening of compounds with structural similarity to BH4, followed by testing with surface plasmon resonance (SPR). Two candidates improved the l-Phe blood concentration. Therefore, given the structural similarity with BH4, it is likely that these compounds act as competitive inhibitors with respect to BH4. Since the identified PCs are recognized or probable inhibitors, at present only the co-factor BH4 can be considered as a non-inhibitory PC.

### 3.2. Congenital Erythropoietic Porphyria

Congenital erythropoietic porphyria (CEP; OMIM #263700), is an ultra-rare autosomal recessive disease affecting 1:1,000,000 persons. CEP is caused by dysfunctions of the uroporphyrinogen III synthase (UROIIIS; EC 4.2.1.75), an enzyme involved in the heme biosynthetic pathway [110,111]. The lack of UROIIIS activity implies the accumulation of type I porphyrins which triggers in turn hemolysis, anemia, splenomegaly and severe phototoxic cutaneous lesions. In most severe cases, premature death can occur in neonatal or early life stages. Until now only symptoms of the disease were addressed and there is currently no approved pharmacological treatment against CEP.

In most cases, CEP is due to *UROS* gene mutations which lead to unfolding, instability and rapid degradation of the mutated UROIIIS protein. Among these mutations (less than 50 according HGMD), the missense p.C73R is the most frequent one (about one-third of reported CEP cases) and the corresponding mutated proteins have an enzymatic stability <1% compared to the wild-type [110].

Recently Millet and coll. performed a full screening combining stability, functional, structural assays, and drug repurposing to identify new UROIIIS PCs. Starting from *in silico* docking of 25,000 compounds on a human isoform of UROIIIS, an allosteric binding site was identified which was targeted by 18.4% of all tested compounds [112]. No mutation reported for CEP involves an amino acid of this allosteric site. Accurate stability, functional and NMR-based structural assays allowed the validation of two fragments targeting this allosteric site. Finally, a drug repurposing study showed that the marketed drug ciclopirox (Table 1) binds to the UROSIII at the allosteric site and is able to restore enzymatic activity *in vitro* and *in vivo*. In 2018, ciclopirox was designated by the US Food & Drug Administration, as well as by the European Medicines Agency and the European Commission in Europe as an orphan medicinal product for the treatment of the CEP.

### 3.3. Methylmalonic Aciduria (MMA) Cdlb Type

Methylmalonic aciduria *cblB* type (MMA *cblB*, OMIM #251110) is an inherited rare disease caused by mutations of the gene (*MMAB*) encoding for the enzyme ATP:cob(I)alamin adenosyltransferase (ATR, EC 2.5.1.17). Patients affected by MMA *cblB* display, during infancy or early childhood, different features including neonatal ketoacidosis, lethargy, failure to thrive and encephalopathy. The estimated incidence of MMA is 1:50,000 to 1:100,000 people. The portion of MMA attributed to *cblB* type is ~13% [113]. ATR is a mitochondrial enzyme involved in coenzyme B12 metabolism. It catalyzes the synthesis of adenosylcobalamin, the cofactor of methylmalonyl-CoA mutase, transferring a 5′-deoxyadenosyl group from ATP to cob(I)alamin (Cbl, vitamin B_12_).

The therapeutic options, in the absence of cure, involves a protein-restricted diet and hydroxycobalamin (OHCbl) supplementation even if less than half of the patients show response to it [114]. Among the 32 different mutations identified in the *MMAB* gene in *cblB* type patients, some expressed mutant proteins display reduced substrate and cofactor affinity (p.R191W), negligible activity and presumed instability *in vivo* (p.R186W, p.R190C, p.R190H and p.E193K), as well as destabilizing mutations that retain some residual activity. More than half of the reported mutations in MMAB *cblB* are missense mutations leading to destabilization and loss of activity of the protein [115,116,117,118]. Thus, pharmacological chaperoning of ATR is a suitable therapeutic option for this misfolding disorder [118,119].

In 2013, Pérez and coll. [119] carried out a DSF-based high-throughput screening to identify compounds that stabilize purified wild-type ATR. For the six compounds identified by this screening, the effect on the stability of wild-type ATR and p.I96T mutant was quantified. The p.I96T protein is enzymatically active with a K_M_ for ATP and K_D_ for cob(I)alamin similar to wild-type enzyme, but exhibits a 40% reduction in specific activity [116]. Compound V (*N*-(((4-chlorophenyl)-carbamothioyl)amino)-2-phenylacetamide, Table 1) was the most effective in stabilizing p.I96T mutant protein and was the only one not to inhibit the specific activity of purified recombinant human ATR at the two tested concentrations (40 and 80 µM).

Molecular docking for compound V identified a most probable binding site located at the C-terminal end of each subunit of the trimeric ATR and adjacent to the binding site of cob(I)alamin but distant from the ATP binding site.

Compound V was also evaluated on a cellular disease model using patient-derived transformed fibroblasts presenting the p.I96T mutation. A significant increase in ATR activity (5.4 fold) was observed after incubation with compound V (80 µM) for 72h. Noteworthy, when OHCbl (1 µg/mL) was co-administrated with compound V, the mutant ATR activity was further improved (~17 fold). This synergistic effect of both substances is supported by the proximity of the binding sites of these two molecules in the docked structure of compound V.

Compound V was also orally administrated to wild-type mice for 12 days and increase of ATR protein level in two disease-relevant organs (liver and brain) supported its stabilizing effect *in vivo*.

More recently, the effect of compound V on six ATR mutants, including the most common (p.R186W) was evaluated. In a cellular model, compound V increased the stability and the activity of all expressed ATR variants and a synergistic effect was also observed with OHCbl [118].

In the same work, Pérez’s group also identified compound VI (4-(4-(4-fluorophenyl)-5-methyl-1*H*-pyrazol-3-yl)-benzene-1,3-diol) as a potential PC on the same mutants [118] but the non-inhibitory character of compound VI was unclear. Indeed, at low concentration of compound VI (40 µM) an inhibition of the specific activity of ATR was observed while at higher concentration (80 µM) this inhibition appeared reversed [119]. Nevertheless, this work confirms that PCs, alone or in combination with OHCbl, can be a promising therapeutic option for the treatment of MMA Cblb type.

### 3.4. Phosphomannomutase 2 Deficiency

The congenital disorder of glycosylation due to phosphomannomutase deficiency (PMM2-CDG, OMIM #212065) is the most common of *N-*glycosylation disorders. This metabolic disorder is caused by the mutation in *PMM2* gene encoding for phosphomannomutase 2 (PMM2, EC 5.4.2.8) [120]. PMM2-CDG is characterized by a very variable clinical picture which may comprise mild to severe neurological disease, mild to pronounced dysmorphia, and variable involvement of many organs. The prevalence of the pathology is estimated to be as high as 1:20,000 [121]. Currently, there is no curative treatment for patients with PMM2-CDG.

PMM2 is a homodimeric enzyme that catalyzes the isomerization of mannose 6-phosphate into mannose 1-phosphate required for lipid-linked oligosaccharide synthesis. This enzyme requires activation by a sugar phosphate, either α-mannose-1,6-bisphoshate or α-glucose-1,6-bisphoshate [122].

More than 110 mutations of *PMM2* gene causing PPM2-CGD have been reported so far and more than 85% of these mutations are missense (from HGMD). Certain PMM2 folding mutations, despite of producing unstable proteins, preserve some level of catalytic activity, suggesting that PMM2-CDG is a conformational disease [123]. Most patients are heterozygous with two different mutant alleles. The most frequent genotype is by far the association of p.F119L/p.R141H mutations [124].

Based on the role of α-glucose-1,6-bisphoshate as a co-activator of PMM2 activity, Cubellis and coll. showed that this compound also stabilizes several pathological mutants [122,124,125]. Recently, starting from this natural PMM2 ligand, the authors synthetized β-glucose-1,6-bisphoshate. The β-isomer proved also able to stabilize pathogenic mutants (p.F119L and p.V129M) as well as wild-type PMM2 but is, like the α -isomer, a mild non-covalent inhibitor of PMM2 [126].

To date, the only non-inhibitory PC described for PMM2-CDG was identified from a DSF-based HTS [127]. This compound, 1-(3-chlorophenyl)-3,3-bis(pyridin-2yl)urea (Table 1), displays no inhibitory effect on the activity of the wild-type PMM2 at any of the tested concentrations. Its capacity to significantly increase the stability of wild-type enzyme as well as the four tested mutants (p.D65Y, p.P113L, p.R162W, p.T236M) was demonstrated by DSF measurements as well as protein degradation time course analysis. It also showed a positive effect on PMM2 activity in a disease model of cells overexpressing mutations compared with baseline activity of the wild-type PMM2 cell line. This ligand also passed the pharmacochemical quality filters and could be regarded as a starting point for the development of new PCs for PMM2-CDG.

## 4. Conclusions

Twenty years after its formal conceptualization, PC strategy has rapidly evolved toward a promising small organic molecule-based therapeutic option for many conformational diseases. The marketing in 2018 of 1-deoxygalactonojirimycin (Galafold^TM^) against FD demonstrated the full clinical potential of the so-called first-generation PCs.

However, such first-generation PCs are active site-specific chaperones, i.e., competitive inhibitors with respect to the substrate, being used at a sub-inhibitory concentration. The therapeutic implementation of such drugs requires a complex discontinuous regimen to rescue the enzyme cellular activity without provoking counterproductive inhibition. The emerging development of second-generation chaperones, defined as non-inhibitory PCs, is thus an extremely promising strategy.

Among the 25 conformational diseases due to enzyme misfolding that were tentatively addressed with PC strategy, only eight gave rise to the report of non-inhibitory PCs. Like for the first-generation PCs, the most studied conformational diseases are LSDs and more particularly Gaucher and Fabry diseases. Yet, very encouraging results were also described for less common pathologies.

The finding of small PC molecules devoid of any inhibition towards the target misfolded enzyme remains highly demanding but the promising advances of recent years indicate that this challenge can be met. Further achievements likely imply a deeper understanding of PC mechanisms of action, both at a molecular and a cellular level. Characterization of the allosteric binding sites and development of biological assays for the straightforward quantification of PC efficacy are key steps along this process. Their development would contribute to the easier development new second-generation PCs with strong therapeutic potential. Several successes in the discovery of new non-inhibitory PCs already exist. The repositioning of ciclopirox against CEP is an encouraging approach highlighting the therapeutic potential of these second-generation chaperones.

## Figures and Tables

**Table 1:** Summary of the diseases due to enzyme misfolding for which a non-inhibitor pharmacological chaperone has been described.

	Protein	Second Generation Pharmacological Chaperone			Reference
		Name	Structure	Known Binding Site (Identification Mode)	
Gaucher disease (OMIM# 231000)	Gcase	2-(2-((4-bromophenyl)amino)-2-oxoethoxy)-*N*-(2-(methyl(phenyl)amino)-2-oxoethyl)benzamide (ML266 or NCGC00182186)	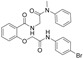	No	[49]
		2-[2-[(4-iodophenyl)amino]-2-oxoethoxy]-*N*-[2- (methylphenylamino)-2-oxoethyl]-benzamide (NCGC607)	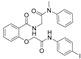	No	[50,51]
		*N*-(4-ethynylphenyl)-5,7-dimethylpyrazolo[1,5-*a*]pyrimidine-3-carboxamide (ML198 or NCGC00188758)	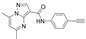	No	[49,52,53]
		5,7-dimethyl-*N*-((1*r*,4*r*)-4-(pentyloxy)cyclohexyl)pyrazolo[1,5-*a*]pyrimidine-3-carboxamide (LTI-291)	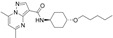	No	[55,56]
		((*S*)-*N*-((2,3-dihydrobenzo[b][1,4]dioxin-2-yl)methyl)-*N*-methyl-2-(pyridin-3-yl)quinazolin-4-amine (S-181)	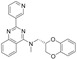	Yes (co-crystallization with a derivatives)	[63,64,65]
Fabry disease (OMIM# 301500)	α-Gal A	2,6-dithiopurine (DTP)	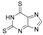	Yes (virtual screening)	[72]
Pompe disease (OMIM# 232300)	GAA	1-(3,4-dimethoxybenzyl)-6-propyl-2-thioxo-2,3,5,6,7,8-hexahydropyrimido[4,5-*d*]pyrimidin-4(1*H*)-one (ML247 or NCGC00183885)	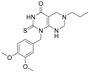	No	[79,80]
		*N*-acetylcysteine	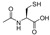	Yes (co-crystallization)	[81,82]
		*N*-butyl-l-deoxynojirimycin (l-NBDNJ)	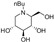	No	[83]
Krabbe disease (OMIM# 245200)	GALC	α-Lobeline	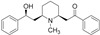	No	[91,92]
		3′,4′,7-trihydroxyisoflavone	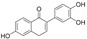	No	[92]
Phenylketonuria (OMIM# 261600)	PAH	(6*R*)-l-erythro-5,6,7,8-tetrahydrobiopterin (BH4)	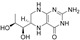	Yes (co-crystallization)	[107,108]
Congenital erythropoietic porphyria (OMIM# 263700)	UORIIIS	Ciclopirox	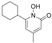	Yes (virtual screening, NMR-based experiments)	[112]
Methylmalonic aciduria *cblB* type (OMIM# 251110)	ATR	*N*-(((4-chlorophenyl)carbamothioyl)amino)- 2-phenylacetamide	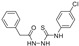	Yes (molecular docking)	[118,119]
Phosphomannomutase 2 deficiency (OMIM# 212065)	PMM2	1-(3-chlorophenyl)-3,3-bis(pyridin-2-yl)urea	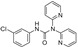	No	[127]
